# Targeting CCL5 signaling attenuates neuroinflammation after seizure

**DOI:** 10.1111/cns.14006

**Published:** 2022-11-28

**Authors:** Zhuoran Zhang, Yan Li, Shihe Jiang, Fu‐Dong Shi, Kaibin Shi, Wei‐Na Jin

**Affiliations:** ^1^ China National Clinical Research Center for Neurological Diseases Beijing Tiantan Hospital, Capital Medical University Beijing China; ^2^ Department of Neurology Tianjin Medical University General Hospital Tianjin China

**Keywords:** CCL5, CCR5, maraviroc, seizure, spatial transcriptomics

## Abstract

**Background:**

Epilepsy is a neurological condition that causes unprovoked, recurrent seizures. Accumulating evidence from clinical and experimental studies indicates that neuroinflammation exacerbates seizure activity.

**Methods:**

We investigated the transcriptional changes occurring in specific brain domains of a seizure mouse model, using 10× Genomics spatial transcriptomics. Differential gene expression and pathway analysis were applied to investigate potential signaling targets for seizure, including CCL5/CCR5 pathway. Maraviroc, an FDA‐approved C‐C chemokine receptor 5 (CCR5) antagonist, was used to verify the impact of CCL5/CCR5 signaling in seizure mice.

**Results:**

We found distinguished regional transcriptome features in the hippocampus of seizure mice. The hippocampus exhibited unique inflammatory gene signatures, including glia activation, apoptosis, and immune response in seizure mice. Especially, we observed notable expression of C‐C chemokine ligand 5 (CCL5) throughout the entire seizure hippocampus. Blockade of CCL5/CCR5 signaling via maraviroc prevented microglia activation and neuron degeneration in seizure mice.

**Conclusions:**

This study supports the potential of CCL5/CCR5 signaling for targeting neuroinflammation after seizure.

## Graphical abstract

The spatial transcriptome demonstrated that the expression level of CCL5 in the hippocampus of KA‐induced seizure mice model was significantly increased. Under the treatment of maraviroc, the reduction of seizure behavior, neuronal degeneration, and glial cell activation were observed, and the survival rate of seizure mice was improved.

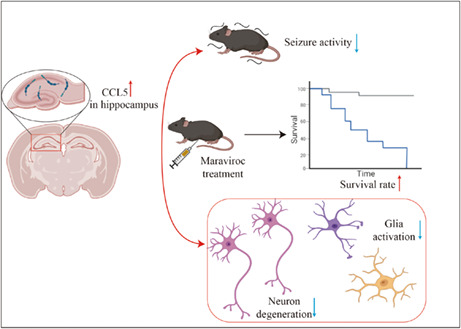

## 1 INTRODUCTION

Epilepsy, which affects approximately 50 million people worldwide, is characterized by an enduring predisposition to generate seizures.^1^ Due to the absence of clear indicators for an impending seizure, seizure is considered more destructive than status epilepticus.^2^ Despite the availability of a wide range of antiepileptic drugs (AEDs), about one‐third of people are refractory to conventional interventions, and the pathogenesis and progression associated with seizures remain poorly understood, resulting in a serious social burden.^3^ Thus, elucidating the mechanisms that are involved in the generation of seizures should aid in the development of novel drugs that modify the seizure process.

The cellular and molecular characteristics of neuroinflammation can be observed in brain slides from seizure patients.^4^, ^5^ Increasing evidence is emerging that neuroinflammation can act both as a consequence and cause of seizure.^6^ Related studies explain the positive effects of anti‐inflammatory treatment on seizures and drug‐resistant epilepsy.^7^, ^8^ Experimental studies have shown that seizure activity can induce neuroinflammation and that recurrent seizures perpetuate chronic inflammation.^9^ In a seizure mouse model, LPS‐induced neuroinflammation can cause and enhance epileptogenesis,^10^ and it has also been shown that some inflammatory mediators are upregulated with seizure activity.^11^, ^12^ For instance, interleukin‐1β (IL‐1β) and its receptor IL‐1R1 have been reported both in human epilepsy foci and in experimental models of seizures and epilepsy.^13^, ^14^ Local injection of IL‐1β antagonists or inhibition of IL‐1β expression in glial cells attenuates kainic acid (KA)‐induced seizures and convulsions.^15^ There are similar reports of antagonist application reducing seizures for TGF‐β, TLR4, and COX families.^16^, ^17^, ^18^ Thus, neuroinflammation is a key regulator of seizure activity, and targeting neuroinflammatory factors, especially chemokines, could be an effective option for the treatment of seizure.

The C‐C motif chemokine ligand (CCL) family is one of the core contributors to neuroinflammation, and it plays a pivotal role in seizure progression. As previously reported, CCL5 is an upregulated chemokine in the hippocampus and other temporal lobe structures in patients with seizure activity,^19^ as are CCL2, CCL3, and CCL4.^20^ These cytokines regulate neural plasticity, vascular permeability, angiogenesis, and immune responses.^21^ The alterations in neuroplasticity, neuroinflammation, and neurovascularity provide a substrate for hyperexcitable neural networks.^22^, ^23^, ^24^ Chemokines interact with chemokine receptors to exert their biological function. Inhibition of C‐C chemokine receptor 5 (CCR5) protected rats from seizures and facilitated neurogenic repair,^25^, ^26^ which underscores the important role of these ligands, including CCL2, CCL4, and CCL5.

To improve understanding of the immunopathogenesis associated with seizures, we explored the structural distribution of glia activation in the brains of seizure mice. Increasing evidence indicates that glia‐derived proinflammatory molecules reduce the seizure threshold and promote seizure onset in the hippocampus of mouse model and patient with temporal lobe epilepsy.^27^, ^28^, ^29^ Thus, we identified differentially expressed genes in the hippocampus of seizure mice. Findings show that CCL5 was highly expressed in the hippocampus of seizure mice. Finally, an FDA‐approved CCR5 antagonist, maraviroc, was used to perform therapeutic intervention in seizure mice.^30^ These results have enriched understanding of the inflammatory feature in seizure, mapped brain region‐specific gene expression profiles, and provided a new view on the immune intervention of seizure activity.

## 2 MATERIALS AND METHODS

### 2.1 Experimental mice

All studies were performed in adult 3‐ to 4‐month‐old male mice. Male C57BL6 mice were purchased from Vital River Laboratory. All mice were maintained under pathogen‐free conditions and housed with no more than five animals per cage with standardized light‐dark cycle conditions and ad libitum access to food and water. All experimental procedures were conducted in accordance with the Animal Research: Reporting of In Vivo Experiments (ARRIVE) guidelines^31^ and approved by the Committee on the Ethics of Animal Experiments of Beijing Tiantan Hospital.

### 2.2 Seizure mice model

To induce seizure activity, mice were anesthetized with 1–1.5% isoflurane at 1–1.5 L/min flow rate and first implanted with EEG electrodes through stereotaxic surgery. After 1 week of recovery, we recorded baseline EEG activity for 30 min in free‐moving mice. Then, we unilaterally injected 0.7 μg (dissolved in 0.35 μl normal saline) of kainic acid (KA) into the hippocampus following coordinates from bregma (AP = −1.5 mm, ML = 1.8 mm, dV = −2.3 mm) via a 10 μl Hamilton microsyringe system. The injection process was controlled by a syringe pump at a rate of 0.11 μl/min. KA‐induced seizure appeared within 30 min after injection, and the degree of seizure activity was measured using the Racine scale. After high‐level seizure activity (higher than grade III) lasting 30 min, 5 mg/kg diazepam was used to relieve spasming by intraperitoneal injection. EEG recordings were terminated after diazepam injection once we observed a 30 min EEG tracing similar to baseline.

### 2.3 Seizure behavior test

Behavior tests were performed immediately after KA injection by two investigators who were blinded to the experimental group assignment. The Racine scale was used to evaluate seizure activities as described before^32^: grade 0 (non‐convulsive); grade I (immobility); grade II (mouth and facial movement); grade III (forelimb clonus and extension); grade IV (clonus in both posterior limb and forelimb); grade V (generalized tonic‐clonic seizure or falling with forelimb clonus). Only mice with high‐level seizure activity (higher than grade III) lasting 30 min were included in experimental group.

### 2.4 EEG recording

EEG recording in a seizure mouse model has been well described.^33^ Seizure activity was considered to have occurred with appearance of isolated or clustered (3–5 Hz, 4–10 s) high‐voltage sharp waves (200–400 μV) and high‐frequency plus low‐voltage electrical activities (10–14 Hz, 60–100 μV) were considered. EEG signals from seizure mice were collected using a Medusa electrophysiological recording system (Bio‐Signal Technologies) and Athena signal acquisition system (Bio‐Signal Technologies) following the manufacturers' instructions. Two independent researchers proofread EEG results.

### 2.5 Immunofluorescence (IF)

Immunofluorescence staining was performed as previously described.^34^ Briefly, frozen brain sections (8‐μm thickness) were made and then permeabilized and blocked in 5% donkey serum consisting of 0.3% Triton X‐100 for 1 h at room temperature. Primary antibodies were diluted by blocking buffer and incubated with sections for 16 h at 4°C. After being incubated with secondary antibodies, nuclei were stained by DAPI (4,6‐diamidino‐2‐phenylindole) and observed by Nikon DS‐Ri2 microscope (Nikon).

### 2.6 Flour‐Jade C (FJC) staining

We detected neuronal degeneration, a characteristic pathological change of seizure, by FJC staining.^35^ Ready‐to‐Dilute (RTD)™ Fluoro‐Jade® C Staining Kit (Biosensis) was chosen for neuronal degeneration detection and used according to the manufacturer's instructions. Briefly, frozen sections were rehydrated and permeabilization via ethanol and sodium hydroxide for 5 min. After potassium permanganate incubation for 7 min to reduce fluorescent background, FJC staining solution was added to sections for 10 min and incubated in the dark. The slides were then cover slipped with DPX and observed via Nikon DS‐Ri2 microscope (Nikon).

### 2.7 Spatial transcriptomics

#### 2.7.1 Tissue preparation

Seizure mice were sacrificed by anesthesia, and fresh brain tissues (without perfusion) were concurrently frozen and then embedded in optical cutting tissue (OCT) compound over Drikold. The quality of OCT‐embedded blocks was reflected in RNA quality, and only tissues with an RNA integrity number (RIN) over 7 were used for library construction.^36^ Brain tissue optimization was performed by using The Visium Spatial Tissue Optimization Slide & Reagent kit (10× Genomics) according to manufacturer's instructions (CG000238). Briefly, seven brain tissue sections were placed on the capture areas on Visium Tissue Optimization slide. After fixing and staining, the tissues were permeabilized for different times, during which released mRNA was bound to oligonucleotides on the Capture Areas. Fluorescent cDNA was synthesized and imaged on the slide. The permeabilization time that results in maximum fluorescence signal with the lowest signal diffusion is optimal. In the present research, all samples were permeabilized for 18 min.

#### 2.7.2 Visium sequencing libraries preparation and sequencing

The Visium Spatial Gene Expression Slide & Reagent kit (10× Genomics) was used to construct sequencing libraries according to the Visium Spatial Gene Expression User Guide. A 10 μm frozen tissue section was placed on one of the Visium gene expression slide capture areas. After tissue hematoxylin and eosin (H&E) staining, bright‐field images were acquired as described in the spatial transcriptomics procedure. Tissue permeabilization was performed for 18 min, as established in the tissue optimization procedure. Then, reverse transcription was conducted and sequencing libraries were prepared following the manufacturer's protocol. Sequencing was performed with a NovaSeq PE150 platform according to the manufacturer's instructions (Illumina) at an average depth of 300 million read pairs per sample.

#### 2.7.3 Visium data analysis

We used an in‐house script to perform basic statistics on raw data and evaluate the data quality and GC content along the sequencing cycles. Raw FASTQ files and histology images were processed with the Space Ranger (version spaceranger‐1.2.0, 10× Genomics) software with default parameters. The filtered gene‐spots matrix and the fiducial‐aligned low‐resolution image were used for downstream data analysis. We used the Seurat package to perform gene expression normalization, dimensionality reduction, spot clustering, and differential expression analysis. Briefly, spots were filtered for a minimum detected gene count of 100 genes. Normalization across spots was performed with the SCTransform function, and 3000 highly variable genes were selected for principal component analysis. For spot clustering, the first 20 PCs were used to build a graph, which was segmented with a resolution of 0.5. Wilcox algorithm was used to perform differential gene expression analysis for each cluster via FindAllMarkers function. In particular, gene expression across brain regions and possible intercellular differences were normalized by sham mouse. By using the differentially expressed genes in the brain of wildtype mice as controls, we extracted the existing differentially expressed genes and hid these results in the KA animals. We adapted Filter protocol in Loupe Browser software and R langrage to do so. We performed a sham sample with normal saline injection as a negative control. Any variation genes in this sample (bottom line) were considered as non‐seizure induced differential expression. Both sham and seizure sections were loaded in Loupe browser, and we used the “exclude” function in Filter to normalized gene expression in seizure brain. For highlighting the variation gene in seizure mouse, we used “setdiff(x,y)” function in R langrage. And the clusterProfiler R package was used for subsequent KEGG and GO analysis.

### 2.8 Flow cytometry

Single‐cell suspensions were prepared from the spleen or the brain as previously reported^37^ and stained with fluorochrome‐conjugated antibodies. Flow cytometry data were collected with a FACSAria III (BD Biosciences) and analyzed by FlowJo 7.6 software (Informer Technologies).

### 2.9 TdT‐mediated dUTP nick end labeling (TUNEL) staining

TUNEL analysis was performed using an In Situ Cell Death Detection Kit, POD (Roche, Basel, Switzerland) according to manufacturer's instructions. Briefly, brain sections were hydrated via deionized water and then permeabilized by 0.3% Triton X‐100 in TBS. TdT and fluorescein‐labeled dUTP were mixed to form the TUNEL staining solution, which was then incubated with brain slides for 20 min at 37°C in the dark. The results were observed via Nikon DS‐Ri2 microscope (Nikon, Tokyo, Japan).

### 2.10 Maraviroc treatment

Maraviroc was purchased from MedChemExpress (MCE). Mice were administered 50 mg/kg of maraviroc or vehicle. Maraviroc was dissolved in solvent (10% DMSO, 40% PEG300, 5% tween‐80 and 45% saline). For preventive treatment, maraviroc and vehicle were injected intraperitoneally (I.P.) once daily for 7 days before model induction. To verify the treatment effect, mice were treated with maraviroc immediately after model induction by intraperitoneal injection at 50 mg/kg or vehicle once daily for 3 days.

### 2.11 Depletion of CCL5 in seizure mice

CCL5 interferential analysis was performed via Entranster™‐in vivo (Engreen Biosystem) according to manufacturer's instruction and previous reports.^38^ Briefly, 2.5 μg small interfering RNA (siRNA) was blended with Entranster™‐in vivo at a ratio of 2:1 and 5% glucose was chosen as diluent to make sure the total volume was 5 μl. The stereotaxic coordinates were as follows: 0.6 mm posterior to the bregma, 1.0 mm lateral to the midline, and 2.5 mm under the dura. One day post‐injection, seizure activities were induced by KA injection. The siRNA used in current study was embellished by methylation plus with cholesterol (RiboBio, Guangdong, China), which could be better used for genetic interference in vivo. The depletion of CCL5 was determined 72 h after seizure onset using qRT‐PCR analysis.

### 2.12 Quantitative real‐time PCR (qRT‐PCR)

Quantitative real‐time PCR was performed as previously described. Total RNA was extracted from mice hippocampus with TRIzol Reagent (Thermo Fisher Scientific) and a total RNA isolation kit (Qiagen). Then, cDNA was synthesized with a SuperScript III First Strand cDNA Synthesis kit (Invitrogen) and analyzed by normalizing the expression of the gene of interest to GAPDH. Quantitative real‐time PCR was performed using SsoAdvanced SYBR Green Supermix (Bio‐Rad) on the CFX96 Real‐time PCR Detection System (Bio‐Rad). The primers we used in the current study are as follow:

CCL5: Forward: GCTGCTTTGCCTACCTCTCC; Reverse: TCGAGTGACAAACACGACTGC. GAPDH: Forward: CAATGACCCCTTCATTGACC; Reverse: GACAAGCTTCCCGTTCTCAG.

### 2.13 Statistical analysis

Animals were randomly assigned to treatment conditions. Data analysis was conducted by investigators who were blinded to the experimental groups. No statistical methods were used to predetermine sample sizes, but the sample sizes were similar to those reported in previous publications.^34^ All statistical analyses were implemented using GraphPad Prism 8 Software. All values are shown as mean ± standard deviation (SD) of three independent experiments. All data used for analysis were normally distributed using Shapiro‐Wilk test. Student's *t*‐test or two‐way ANOVA was performed to evaluate pairwise comparison or multivariate analysis. All statistical analyses were performed using SPSS statistics 22 software. *p* value <0.05 was judged to be statistically significant.

## 3 RESULTS

### 3.1 Activation of microglia and astrocytes in the hippocampus after seizure activity

**FIGURE 1:**
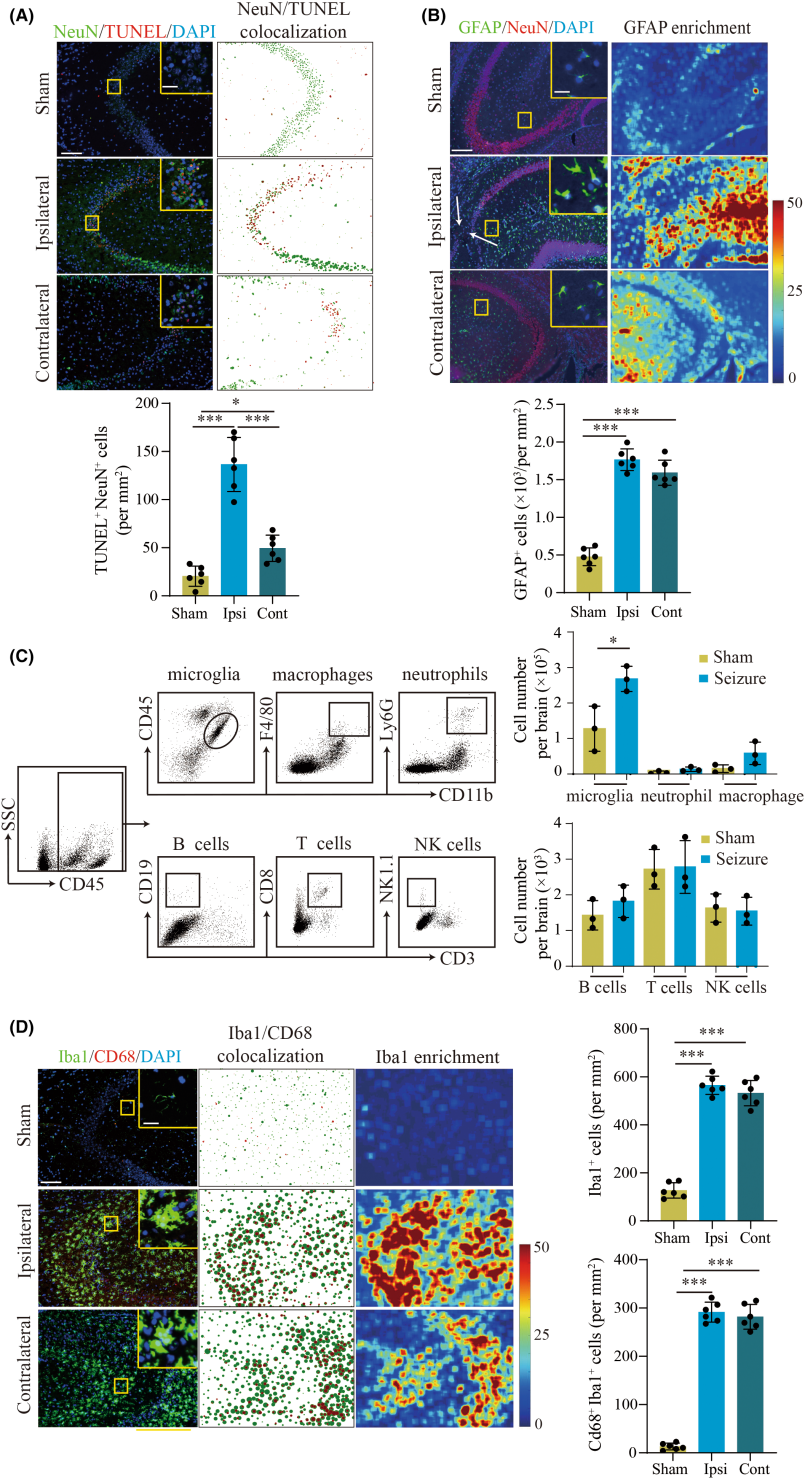
Microglia and astrocyte activation in the hippocampus of mice at day 3 after seizure induction. A. TUNEL analysis showing neuron apoptosis in the hippocampus. Left: representative immunofluorescence staining of TUNEL (red) and NeuN (green). Scale bar, 100 μm (Insert: 20 μm). Right: particle co‐expression analysis showing neuron apoptosis. Statistical graphs: Quantitative analysis of TUNEL‐positive neurons. B. Immunofluorescence staining of GFAP and NeuN indicated the cell number of astrocytes and neurons in seizure hippocampus, including ipsilateral (Ipsi) and contralateral (Cont). Left: representative images of immunofluorescence staining of GFAP (green) and NeuN (red). Scale bar, 100 μm (Insert: 20 μm). Right: Fluorescence heatmap analysis of astrocytes in the hippocampus of seizure mice compared to sham mice. White arrows point to neuronal loss. Bar graphs show quantitative analysis of GFAP^+^ cell counts. C. Flow cytometry (FCM) analysis of immune cell infiltration in the brains of seizure mice. Left: FCM gating strategy of microglia, macrophages, neutrophils, B cells, T cells, and NK cells. Right: quantitative analysis of FCM. D. Immunofluorescence staining of activated microglia (Iba1^+^ CD68^+^) in the hippocampus. Left: representative images of immunofluorescence staining of Iba1 (green) and CD68 (red). Scale bar, 100 μm (Insert: 20 μm). Middle: particle analysis of Iba1 and CD68 co‐localization in microglia. Right: fluorescence heatmap of Iba1 showed the aggregation of microglia in bilateral hippocampus, especially the CA3 region. Statistical graphs: quantitative analysis of Iba1^+^ cell and CD68^+^ Iba1^+^ cell. *n* = 6 mice for each group in A, B, and D; *n* = 6 mice, each group containing 3 mice in C. Mean ± SD. *p < 0.05, **p < 0.01, ***p < 0.001 by Student's *t*‐test

A seizure mouse model was induced by kainic acid (KA) injection (Figure S1A) and the representative characteristics were used to describe seizure activities, including electroencephalogram (EEG), behavior test, and FJC staining (Figure S1). Typical neuronal cell loss and apoptosis were confirmed in both ipsilateral and contralateral hippocampus, the predominant region of seizure origin and maintenance, at day 3 after KA injection (Figure 1A,B). To better understand the immune basis of seizure progression, we screened for the prevalence of brain resident immune cells and brain‐infiltrated leukocytes after seizure activity. As shown in Figure 1C, we found a significant increase in CD45^int^CD11b^+^ microglia in seizure mice. We confirmed that microglia and astrocytes were activated in both ipsilateral and contralateral hippocampus (Figure 1B,D). These data show dramatic immune activation in the hippocampus in association with seizure progression.

### 3.2 Seizure induces distinguished changes to the spatial transcriptome features of the hippocampus

**FIGURE 2:**
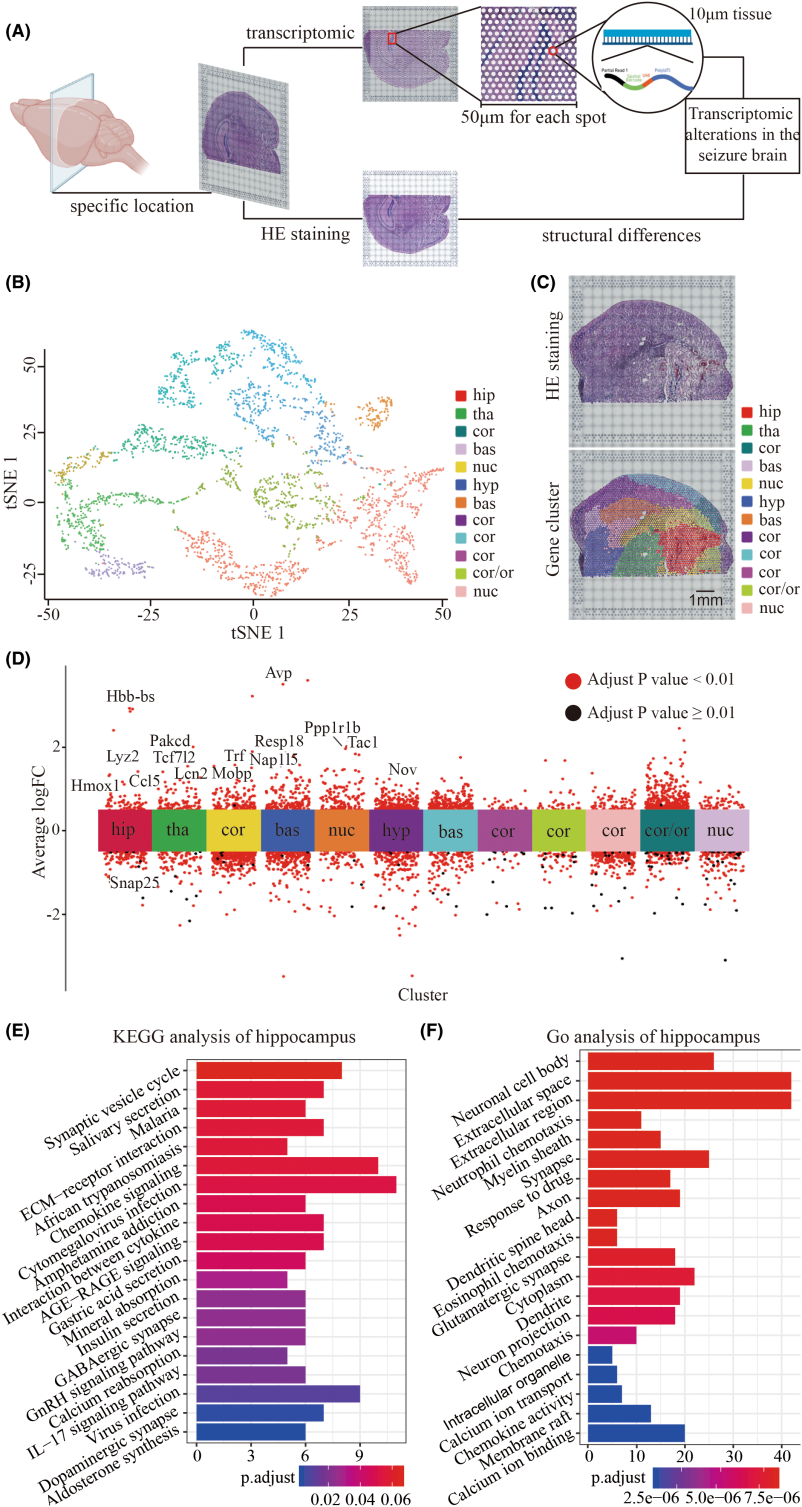
Emergence of distinctive transcriptomic profiles in the hippocampus of seizure mice. A. Schematic diagram showing the technical route of Visium transcriptome for seizure mice. B. tSNE cluster analysis of seizure mice identified structure‐related differential expression gene. C. Upper: hematoxylin‐eosin (HE) staining revealed the anatomical structure of a mouse brain. Lower: gene expression clusters. The annotation of each cluster is based on HE staining of the anatomical structure, including the hippocampus, cortex, basal, thalamus, hypothalamus, and nucleus. Each color represents one gene cluster. Scale bar, 1 mm. D. Differentially expressed gene (DEG) analysis of seizure mice showed differences in gene expression. The gene clusters are represented by each color, as shown in the panel's legend. Adjusted p value less than 0.01 was considered significant. E‐F. KEGG and GO analysis of DEGs in the hippocampus. bas, basal; cor, cortex; hip, hippocampus; hyp, hypothalamus; tha, thalamus

To further explore the spatial transcriptome features of the hippocampus as compared to other anatomical structures, we performed Visium transcriptome sequencing on the brains of seizure mice (Figure 2A).^39^ We observed distinguished changes to the gene expression profile in brains of seizure mice (bregma −1.94 mm). We generated a tSNE map of 3115 spots from seizure mouse (Figure 2B), and we identified 12 clusters based on anatomical structure (Figure 2C). These clusters could be grouped into six main anatomical categories: (1) cortex; (2) hippocampus; (3) thalamus; (4) hypothalamus; (5) basal and (6) nucleus (Figure 2B,C). Compared with other anatomical structures, the upregulated genes in the hippocampus, including CCL5, Lyz2, and HMOX1, were mainly related to immune response (Figure 2D), suggesting that the hippocampus may acquire pro‐inflammatory features in seizure model. Similarly, KEGG analysis revealed 20 enriched pathways in the hippocampus, especially chemokine signaling pathways, interactions between cytokines, and cytokine and extracellular matrix (ECM) receptor interaction (Figure 2E). Additionally, the GO database gave evidence of signal transduction in the cellular microenvironment, including chemotaxis and extracellular region (Figure 2F).

**FIGURE 3:**
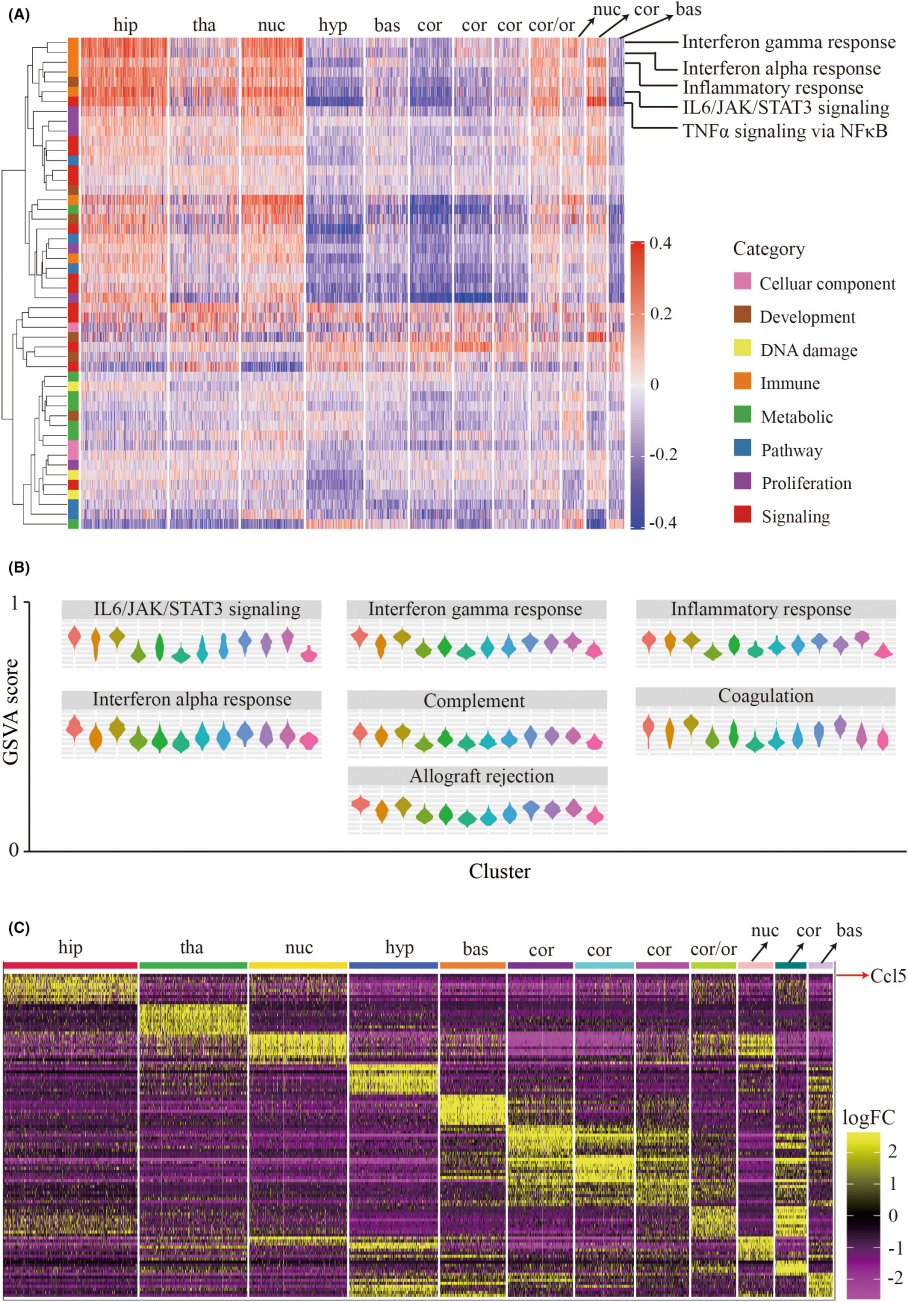
Biological function analysis revealed the enrichment of CCL5‐related signaling in seizure hippocampus. A. Heatmap showing enrichment of biological functions for each anatomical cluster via GSVA analysis. Biological functions include cellular components, DNA damage, metabolic, proliferation, development, immune, pathway, and signaling. B. Violin graphs showing major immune‐related processes in each anatomical structure. C. Heatmap indicates top 10 DEGs in each cluster. Red arrow means that CCL5 was the most highly expressed gene in the hippocampus compared to other structures. Abbreviation: hip, hippocampus; cor, cortex; hyp, hypothalamus; bas, basal; tha, thalamus

Gene set variation analysis (GSVA) score showed a significant upregulation in inflammation‐related pathways (interferon response, IL‐6/JAK/STAT3 signaling, and TNF‐α signaling) in the hippocampus (Figure 3A,B), suggesting the pivotal role of the hippocampus in the pro‐inflammatory status after seizure. Among all the differentially expressed genes (DEGs), CCL5 exhibited the highest expression level in the hippocampus as compared to other genes (Figure 3C).

### 3.3 Spatiotemporal characterization of the transcriptome in hippocampus of seizure mouse

**FIGURE 4:**
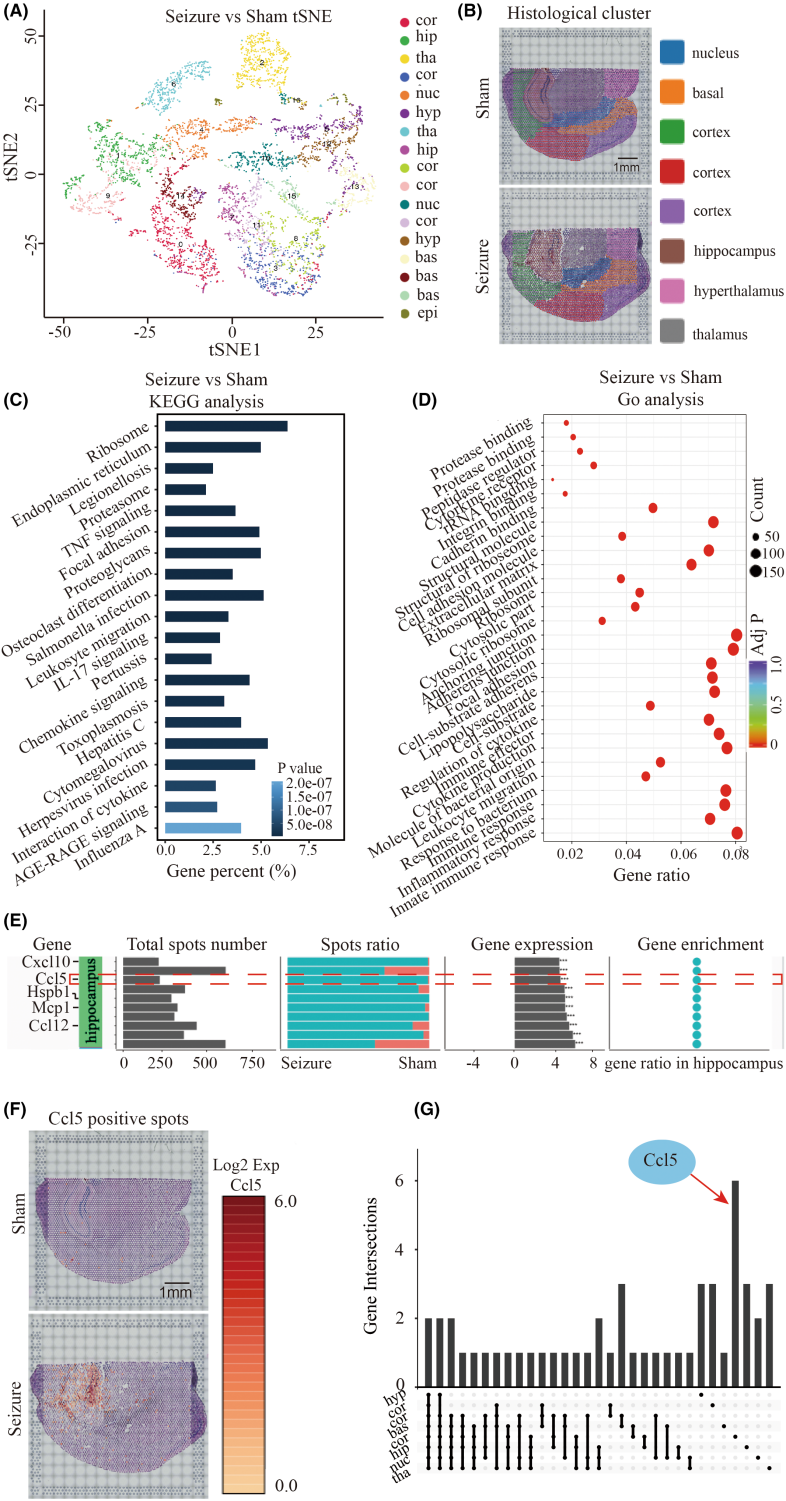
CCL5 is upregulated in the hippocampus of a seizure mouse compared to a sham mouse. A. tSNE analysis of seizure mouse brain comared to sham mouse brain. B. Gene cluster division adapted to anatomical structure. On the basis of differential gene expression distribution, and regions of interest (ROI), regions were divided into the cortex, hippocampus, thalamus, hypothalamus, and nucleus for subsequent analysis. Each color represents a specific anatomical structure. Scale bar, 1 mm. C–D. KEGG and GO analysis of epileptic hippocampus against sham hippocampus. E. Spatial enrichment analysis of the top 10 genes (by expression level) in the hippocampus. The headlines were explained as follows: top 10 upregulated gene names in hippocampus, positive spot number of each gene in both sham and seizure group, positive spot ratio of each gene in seizure mouse compared to sham mouse, gene enrichment in hippocampus. F. The enrichment of CCL5 in epileptic and sham brain. The red highlighted spots represent CCL5 expression at the modified position. Scale bar, 1 mm. G. Venn diagrams showing subsets of gene expression

We next focused on transcriptomic differences in the hippocampus between seizure mice and sham mice. A total of 6122 spots in both seizure and sham mice were divided into 17 categories (Figure 4A) that exhibited variation within the same anatomy. To specifically compare the transcriptomic profiles of the hippocampus of seizure and sham mice, we delineated anatomical clusters based on anatomical structures, which is indicated by HE staining (Figure 4B). By screening the differentially expressed genes between the sham and seizure mice in the hippocampus, we found a higher expression of chemokines‐related genes (MCP1, CCL12, and CCL5) and glia‐related genes (LCN2 and SPP1). KEGG analysis showed a higher enrichment in inflammation and chemotaxis‐related pathways, including IL‐17 signaling pathway, TNF signaling pathway, influenza A, and leukocyte migration (Figure 4C). Go analysis was mainly enriched in protein function, cell adhesion, and immune response‐related functions, including protease binding, cytokine production, and immune effector functions (Figure 4D). Notably, CCL5 and CCL5*‐*related genes, such as NF‐κB, Fos, Ask1, and Traf3, were widely enriched in the above pathways, indicating preferential expression of CCL5 in the hippocampus of seizure mice (Figure 4E). By examining hippocampus‐specific gene clusters, we found that CCL5 had the highest ratio of positive spots in the hippocampus (Figure 4E,F). These findings indicate that CCL5 is a specific gene expressed in the hippocampus following seizure (Figure 4G). Together, our data provide direction for future research on the mechanism of seizure progression and show a potential role for hippocampal CCL5 in seizure progression.

### 3.4 Maraviroc reversed KA‐induced seizure activities

**FIGURE 5:**
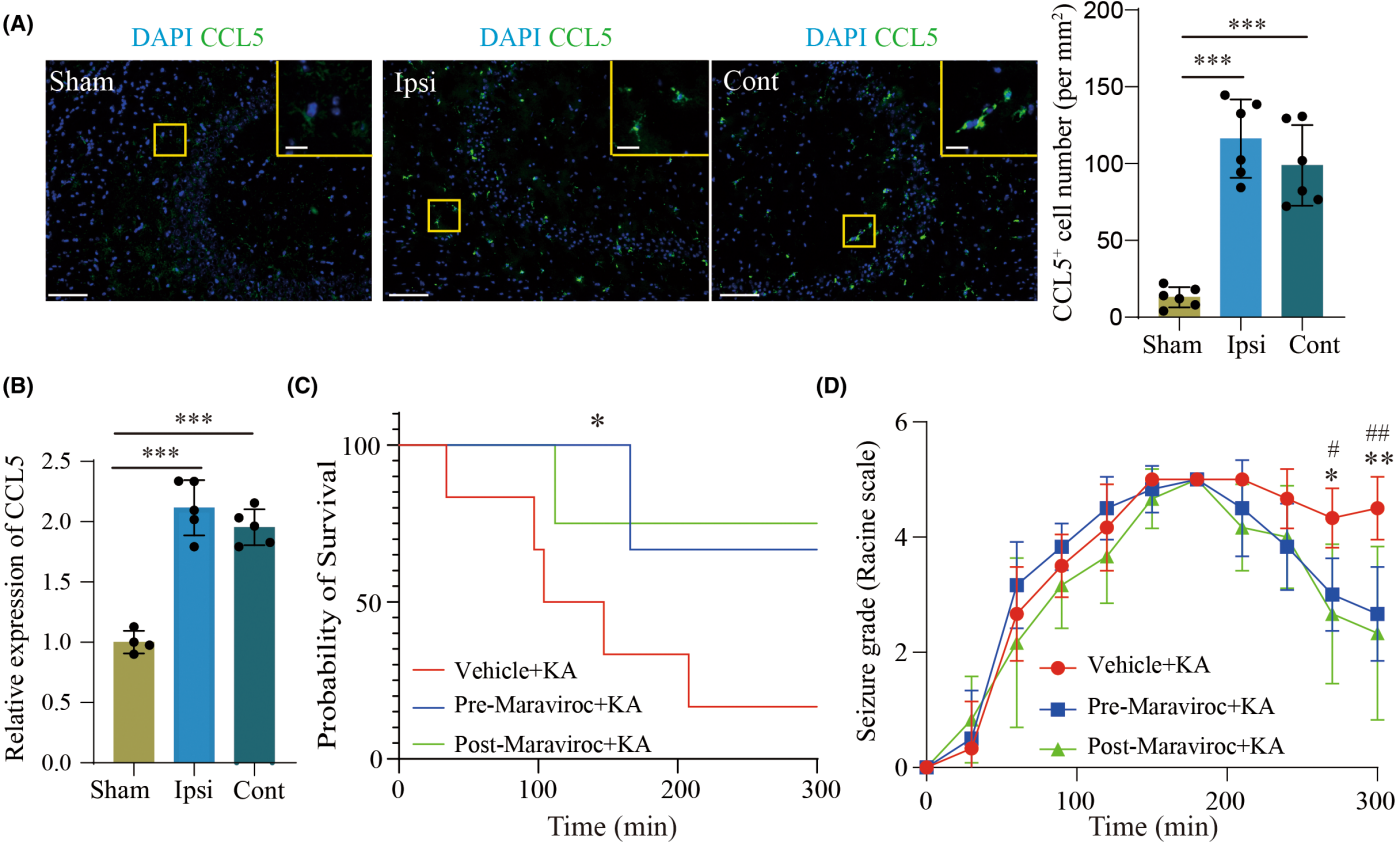
Maraviroc reverses KA‐induced seizure activity in a murine model. A. Immunofluorescence analysis of CCL5 in the hippocampus of seizure mice and sham mice. Scale bar, 100 μm (Insert: 20 μm). B. qRT‐PCR analysis of CCL5 in seizure mice and sham mice. GAPDH was chosen as normalized control. C. Survival analysis of seizure mice under maraviroc or vehicle treatment. D. Seizure activity in seizure mice was measured via Racine scale under maraviroc or vehicle treatment. *n* = 6 for each group. Mean ± SD. *p < 0.05, **p < 0.01, ***p < 0.001 by Student's *t*‐test

**FIGURE 6:**
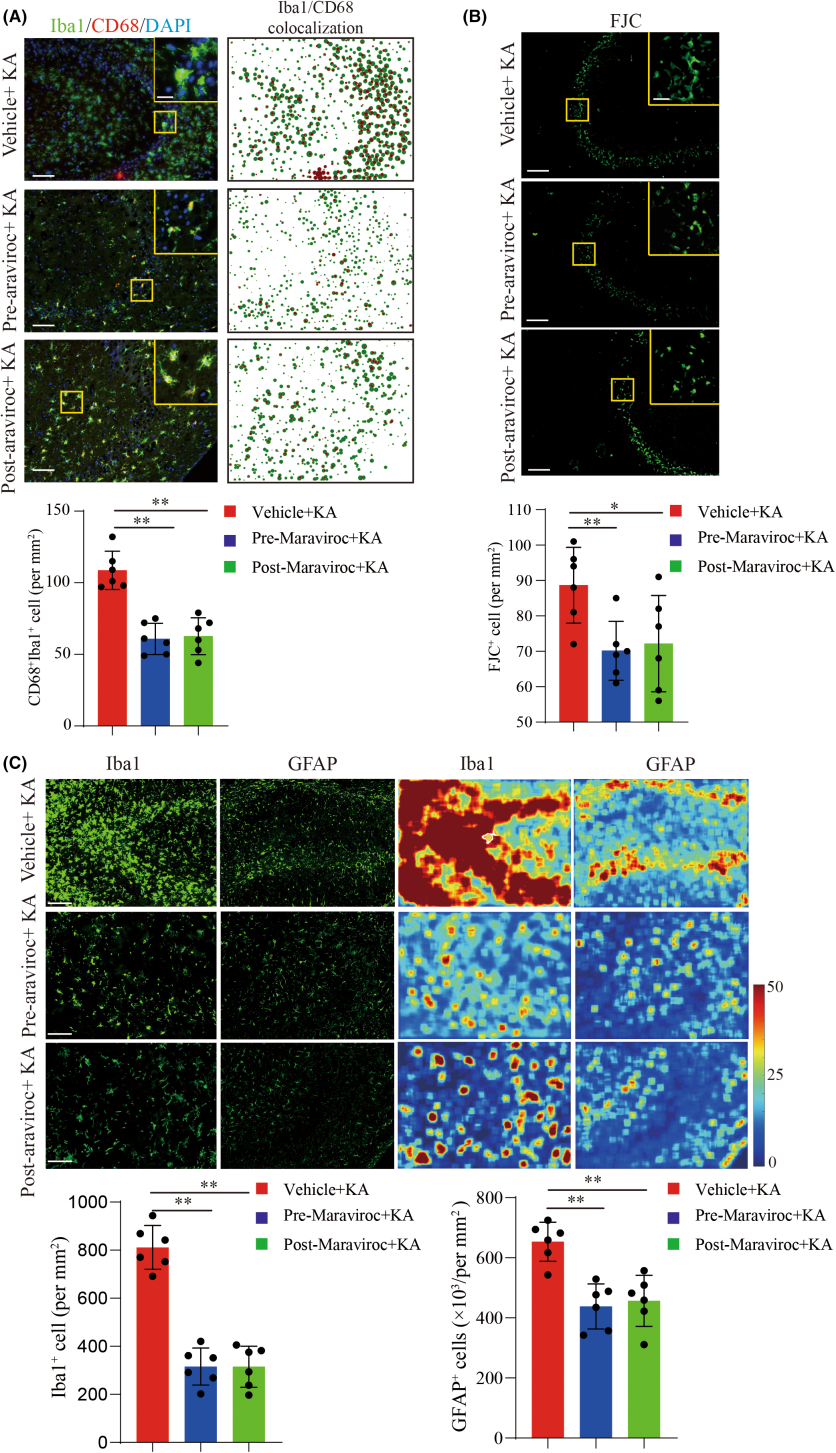
Maraviroc treatment reduced glial cell activation and neuron degeneration in seizure mice. A. Immunofluorescence staining of microglial cell activation after maraviroc treatment in seizure mice. Upper: representative images of immunofluorescence staining of Iba1 (green) and CD68 (red). Scale bar, 100 μm (Insert: 20 μm). Lower: particle analysis of Iba1 and CD68 co‐localization revealed activation of microglial cells. Statistical graphs: quantitative analysis of Iba1^+^CD68^+^ cell. B. Flour‐Jade C (FJC) staining exhibited neuron degeneration in the CA3 region of hippocampus. Upper: representative image of neuronal degeneration, scale bar:100 μm (Insert: 20 μm). Lower: quantitative analysis of FJC staining. C. Top: immunofluorescence staining of Iba1 and GFAP represented the aggregation and activation of microglia and astrocytes. Line 1 contains representative images of immunofluorescence staining of Iba1, corresponding to the fluorescence heatmap in line 3. Line 2 contains representative images of GFAP staining, corresponding to the fluorescence heatmap in line 4. Scale bar, 100 μm. Bottom: the quantitative analysis of Iba1‐positive cells and GFAP‐positive cells. *n* = 6 for each group. Mean ± SD. *p < 0.05, **p < 0.01 by Student's *t*‐test

To further explore the regulatory role of CCL5 in seizure progression, we measured CCL5 expression in the hippocampus of seizure mice brains. As shown in Figure 5A,B, upregulation of CCL5 in ipsilateral hippocampus was confirmed by immunostaining and PCR analysis. We first knocked down CCL5 in the hippocampus of seizure mice (Figure S2A) 24 h before seizure onset via in vivo small interfering RNA (siRNA) injection,^40^ embellished with cholesterol and transfected via EntransterTM in vivo. We found that CCL5 gene silence reduced the intensity of seizure activity and improved survival rate in seizure mice (Figure S2B,C). Corresponding pathological examination exhibited depletion of CCL5, revised neuronal degeneration, and microglia activation (Figure S2D,E). It has been reported that CCL5 induces cell migration via binding to the corresponding receptor, CCR5.^41^, ^42^ We blocked the interaction between CCL5 and CCR5 by using maraviroc,^42^, ^43^ an FDA‐approved CCR5 antagonist, to inhibit the biological function of CCL5. We found that the survival rate of seizure mice was significantly increased after pre‐ and post‐maraviroc treatment (Figure 5C). Meanwhile, seizure activity was significantly suppressed in seizure mice after maraviroc treatment (Figure 5D). Further pathological examination revealed that maraviroc treatment attenuated neuronal degeneration and microglia and astrocyte activation (Figure 6A–C). Taken together, our results suggest maraviroc may be beneficial to neuroinflammation and attenuate seizure pathologies by targeting CCL5/CCR5 signaling.

## 4 DISCUSSION

In this study, we observed CCL5 upregulation in the hippocampus after seizure, by adopting spatial transcriptome to visualize the gene features in different anatomical structures of brain. We provided evidence that genetic knockdown of CCL5 and blockage of CCL5/CCR5 signaling are both beneficial to seizure pathology, including decreased seizure activity, improved survival, attenuated neuronal degeneration, and reduced neuroinflammation (Figure S3). This study reports the first application of maraviroc, an FDA‐approved CCR5 antagonist, in seizure preclinical research.

Neuroimmune regulation is one of the core mechanisms of seizure activity. As reported by Chen and his colleagues, some immune‐related gene, such as Lgals3 and Serpine1, have been identified as hub genes in human temporal lobe epilepsy.^44^ Meanwhile, recent evidence from human studies suggests a link between neuroinflammation and seizure that is supported by the anti‐inflammatory effects of commonly prescribed anticonvulsants.^45^ Significant infiltration of immune cells in resected brain tissues from patients with inherited or acquired seizure has also been identified.^46^ Notably, during the early stages of seizure activity, the main pathological feature is the activation of innate immune cells, including microglia and astrocytes.^47^ Activated microglia and astrocytes release cytokines that induce transcriptional signals within glia or within the microenvironment, resulting in an altered epileptic threshold. Activated microglia can also regulate seizure severity by releasing cytokines.^48^ These findings confirm the impact of immune regulation, especially cytokine regulation, on seizures. Our study identified similar glial activation in the hippocampus and filled the gap of spatial and temporal expression differences via Visium transcriptomics in seizure mice. We focused on the hippocampus structures of seizure mice, which displayed specific genes variation compared to other anatomical structures. Among these differentially expressed genes, CCL5 was the most increased in seizure hippocampus compared to other anatomical structures and sham hippocampus, consistent with previous reports.^20^ Further, CCL5‐positive anatomical structure exhibited highly enrichment on inflammation pathway (Chemokine signaling, IL‐17 signaling, virus infection) and neuronal activity‐related pathway (GABAergic synapse, calcium reabsorption, dopaminergic synapse). These results imply a link between CCL5, inflammatory regulation, and neural activity, which give evidence for the development of seizure therapeutic targets.

Previous studies have reported that the CCL5/CCR5 pathway is involved in multiple types of diseases.^49^ Although a few reports have shown that CCL5 is highly expressed in the hippocampus and piriform cortex of epilepsy and seizure patients,^50^ its regulatory role and mechanism in epileptogenesis are still unknown. Maraviroc is an FDA‐approved CCR5 antagonist that has been successfully used for HIV infection treatment. In recent reports, availability of maraviroc in cerebrospinal fluid in stroke mice has been confirmed, which provided theoretical support for intracranial application of maraviroc in the current study.^30^ It is worth noting that maraviroc can protect against the development of stroke and multiple sclerosis by enhancing neural repair and reducing inflammatory response.^30^, ^51^ Maraviroc currently has no approved application in seizures, and our report is the first study suggesting the use of maraviroc in the early stages of seizure. We observed a reduction in microglia and astrocyte activation, corresponding to neuron apoptosis in seizure mice, which lead to lower mortality and seizure activity. These results expand previous studies and illustrate the potential for attenuating neuroinflammation and seizure pathologies by targeting CCL5/CCR5 signaling. This study has several limitations. We did not deeply explore the mechanism by which CCL5 regulates seizure activity. Furthermore, the biological role of CCL5 in recurrent epilepsy was not further explored, and further investigations are warranted.

## 5 CONCLUSION

In conclusion, this study fills in the gaps in the spatiotemporal differences of gene expression in the brains of seizure mice. It elucidates the contribution of hippocampus‐specific CCL5‐CCR5 signaling in seizure pathology, which may enrich evidence for targeting CCL5 signaling in seizures.

## AUTHOR CONTRIBUTIONS

W.‐N. J. formulated the concept of the study. Z.‐R.Z. and Y.L. performed experiments and analyzed the data. W.‐N. J., K.S., Z.‐R.Z., Y.L., S. J., and F.‐D.S. interpreted the results and drafted the manuscript. All authors read and approved the final manuscript.

## FUNDING INFORMATION

This work was supported in part by National Science Foundation of China (82122021, 81971094, 81771274); National Science Foundation of Beijing (7192059); the Natural Science Foundation of Tianjin Province (18JCYBJC43800).

## CONFLICT OF INTEREST

The authors declare that they have no competing interests.

## CONSENT FOR PUBLICATION

Not applicable.

## Supporting information


Figure S1

Figure S2

Figure S3

Appendix S1

## Data Availability

All data generated or analyzed during this study are included in this published article, its supplementary information files, and are available from the corresponding author on reasonable request.
