# Supplementary figures and images for: Targeting CCL5 signaling attenuates neuroinflammation after seizure
Source: CNS Neurosci Ther. 2022 Nov 28;29(1):317–30. doi: 10.1111/cns.14006 (PMC9804050; doi:10.1111/cns.14006)

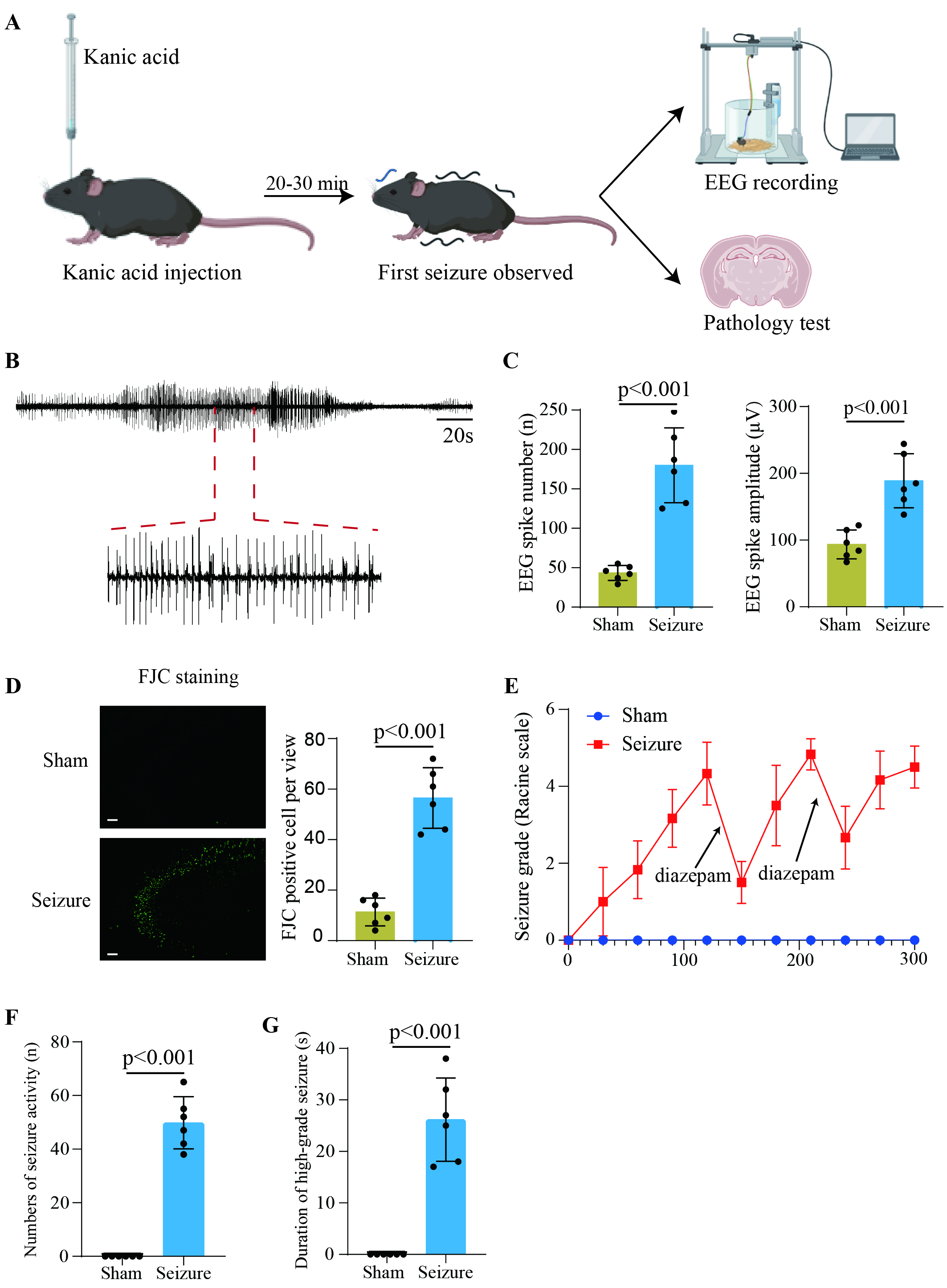

Supplement: Supplementary file 1 — Figure S1 [file CNS-29-317-s002.tif]

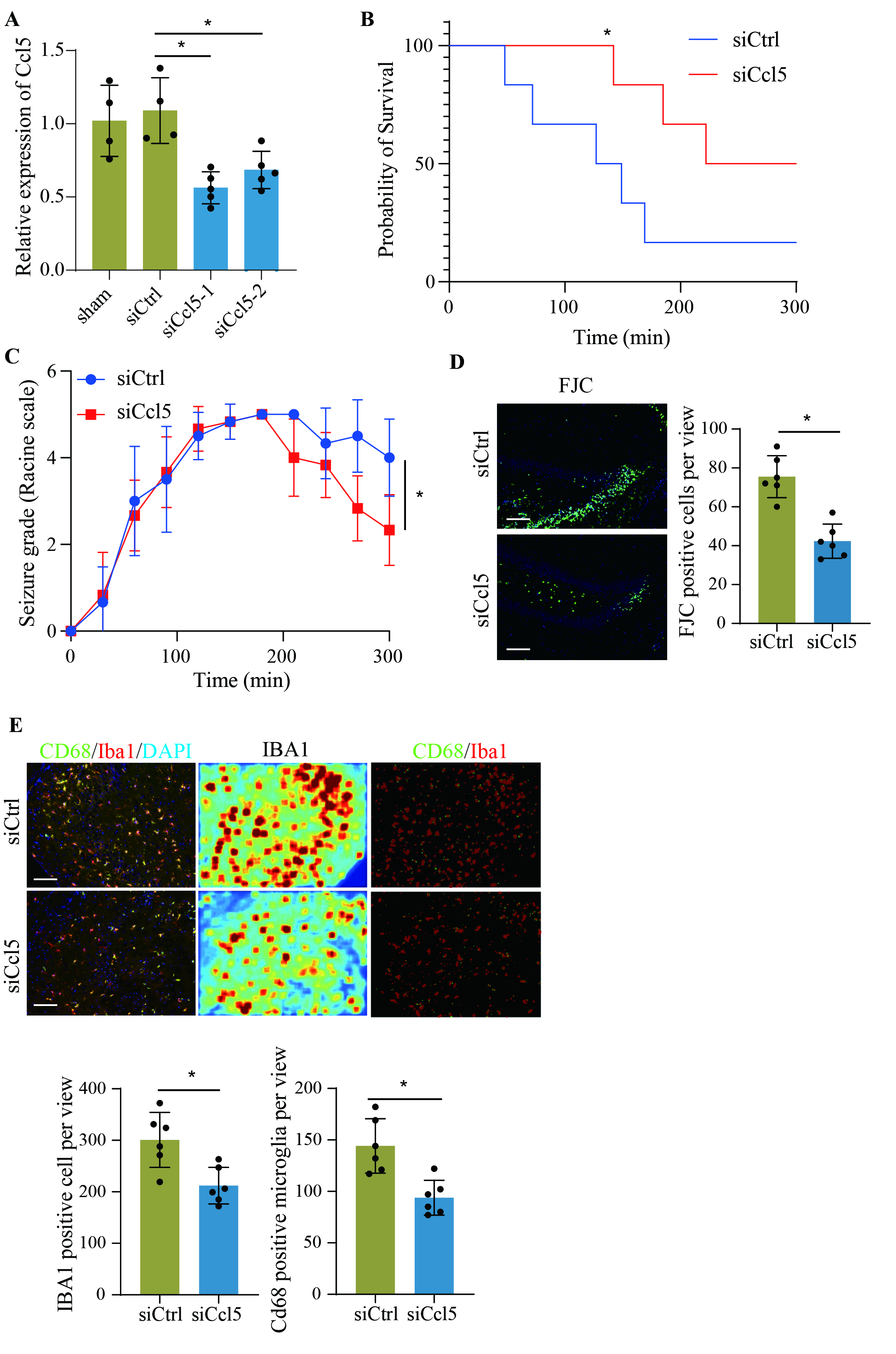

Supplement: Supplementary file 2 — Figure S2 [file CNS-29-317-s003.tif]

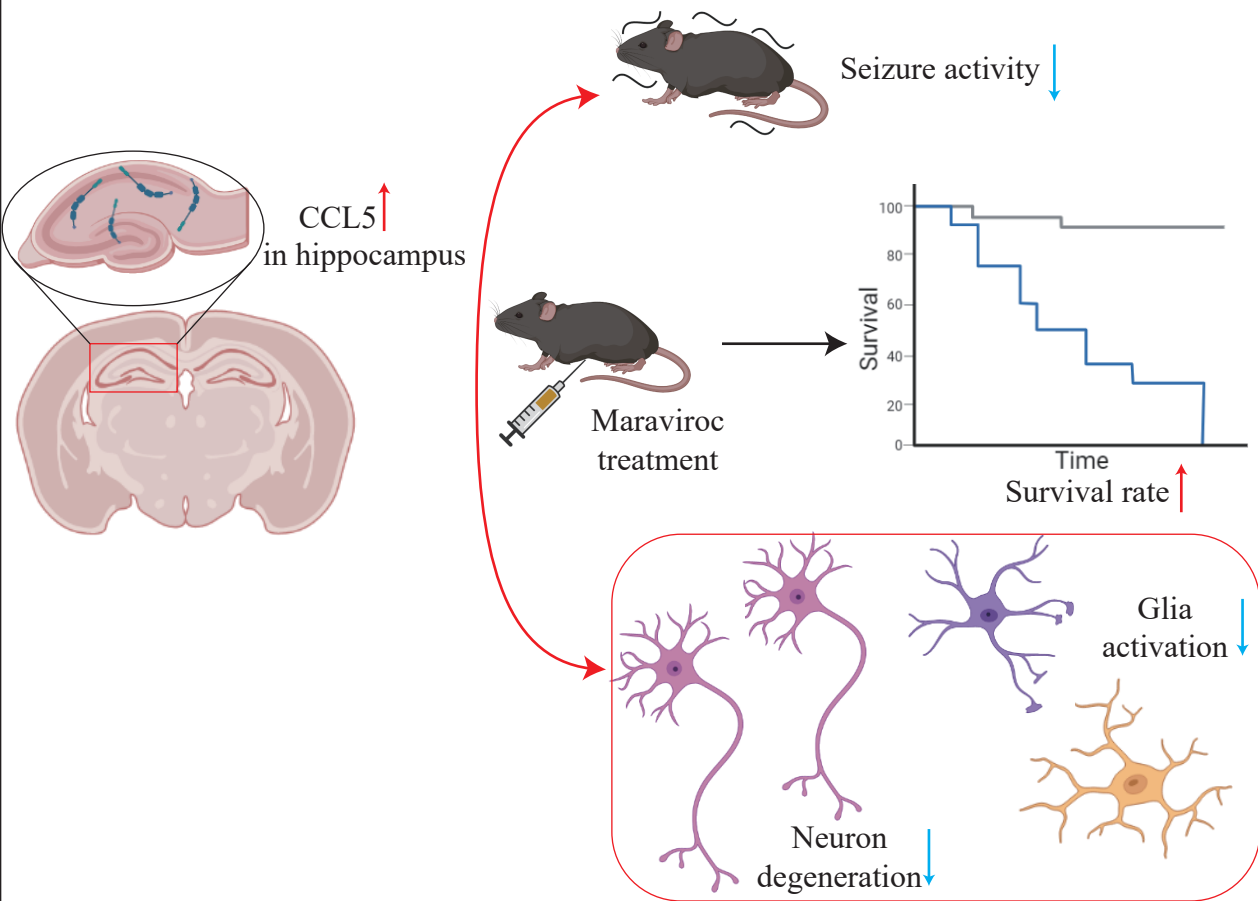

Supplement: Supplementary file 3 — Figure S3 [file CNS-29-317-s004.pdf]
