# Supplementary material for: Targeting CCL5 signaling attenuates neuroinflammation after seizure
Source: CNS Neurosci Ther. 2022 Nov 28;29(1):317–30. doi: 10.1111/cns.14006 (PMC9804050; doi:10.1111/cns.14006)
Supplement: Supplementary file 4 — Appendix S1 [file CNS-29-317-s001.docx]

**Supplementary Figure 1. Hyper-excitability and neuron degeneration in KA-induced seizure mice.**

A. Schematic diagram showing the details of KA-induced seizure mice. Diazepam was not suitable for each mouse and was dependent on the degree of seizure activity. B. Electroencephalogram (EEG) recording showed typical spinous complex wave during epileptic seizures. C. Quantitative analysis of EEG recordings shows a higher electroneurographic signal and frequency in seizure mice. D. Flour-Jade C (FJC) staining exhibited neuron degeneration in the CA3 region of the hippocampus. Left: representative images of neuronal degeneration. Scale bar:100 μm. Right: quantitative analysis of FJC staining. E. Seizure activities were measured via Racine scale after KA-injection. Black arrow indicates spasmolysis by diazepam injection. F-G. Number of spasms and tonic-clonic seizure activity. n=6 for each group in C-G. n=6 for each group. Mean ± SD. Statistical analysis was determined by Student’s *t*-test.

**Supplementary Figure 2. Knockdown of CCL5 leads to better prognosis after seizure.**

A. qRT-PCR analysis exhibited knockout efficiency of CCL5 siRNA. n=4-5 for each group. B. Survival analysis showed higher survival rate after CCL5 depletion. C. Knockdown of CCL5 resulted in lower seizure grade. D. Flour-Jade C (FJC) staining exhibited neuron degeneration in hippocampus. Left: representative image of neuronal degeneration. Scale bar:100 μm. Right: quantitative analysis of FJC staining. E. Immunofluorescence staining of activated microglia (Iba1^+^CD68^+^) in the hippocampus. Left: representative images of immunofluorescence staining of Iba1 (green) and CD68 (red). Scale bar: 100 μm. Middle fluorescence heatmap of Iba1 showed the aggregation of microglia in bilateral hippocampus. Right: particle analysis of Iba1 and CD68 co-localization in microglia. n=6 for each group. Mean ± SD. **P*<0.05 by Student’s *t*-test.

**Supplementary Figure 3. Graphic abstract**
